# Efficacy and safety of the fixed combinations of tafluprost/timolol and latanoprost/carteolol

**DOI:** 10.1038/s41598-019-44028-2

**Published:** 2019-05-16

**Authors:** Masahiro Fuwa, Atsushi Shimazaki, Masafumi Mieda, Naoko Yamashita, Takahiro Akaishi, Takazumi Taniguchi, Masatomo Kato

**Affiliations:** Research and Development, Santen Pharmaceutical Co., Ltd., Ikoma-shi, Nara Japan

**Keywords:** Glaucoma, Pharmacodynamics, Preclinical research

## Abstract

In this study, we made a comparative efficacy and safety assessment of two different fixed combinations of drugs, viz., tafluprost/timolol (TAF/TIM) and latanoprost/carteolol (LAT/CAR), by determining their effects on intraocular pressure (IOP) in ocular normotensive monkeys and examining their toxic effects on ocular surface using human corneal epithelial cells. TAF/TIM was found to be more effective in lowering IOP for a longer duration compared to LAT/CAR. We found that the difference in the intensity of IOP-lowering effect was because of the differences in the strength of timolol compared with that of carteolol as a beta-adrenergic antagonist and strength of tafluprost compared with that of latanoprost as a prostaglandin analogue. In addition, TAF/TIM showed much less cytotoxic effects compared to LAT/CAR on the human corneal epithelial cells. Our findings showed that TAF/TIM is better than LAT/CAR with regard to the IOP-lowering effect in monkeys and toxicity on ocular surface.

## Introduction

Glaucoma is a neurodegenerative disease of the eyes characterised by selective retinal ganglion cell loss, followed by progressive defects in visual field, resulting in the principal cause of irreversible blindness worldwide^1–4^. Elevated intraocular pressure (IOP) is an important contributor for the progression of glaucoma, for which the current treatment primarily involves IOP reduction^1,5–8^.

Initial pharmacological treatment of glaucoma is done using monotherapies, including beta-adrenergic antagonists or prostaglandin analogues (PGAs). PGAs lower IOP by improving uveoscleral outflow, and beta-adrenergic antagonists reduce IOP by decreasing aqueous humor production^4^. Although tafluprost and latanoprost, used in the present study, are PGAs, several differences exist in their structure, profile of the IOP-lowering effect and binding affinity for prostanoid FP receptor^9^. There are two fluorine atoms in the omega-chain of the prostaglandin structure in tafluprost at the 15^th^ carbon atom; these atoms are absent in latanoprost^9^. In comparison with latanoprost, tafluprost has more potent binding affinity for prostanoid FP receptor, which may be because of the fluorine atoms present in the tafluprost structure^9^. These differences may have contributed to the different IOP-lowering effect of tafluprost compared with latanoprost, as found in the present study. As for the differences between beta-adrenergic antagonists, timolol and carteolol, carteolol has an intrinsic sympathomimetic activity and timolol does not, although both are non-selective beta-adrenergic antagonists^10,11^. However, monotherapy is not sufficient in >40% of patients; hence, these patients are often administered ≥2 IOP-lowering drugs^12–16^. A disadvantage of multiple drug therapy is that it involves frequent doses and intricate dosing schedules, making it difficult for patients to properly manage their treatment regimen^17,18^. Thus, there is a concern among clinicians about the IOP-lowering drug efficacy in the patients who do not comply with the suggested dosing instructions.

Fixed combination (FC) therapy is helpful for achieving compliance by the patients^19^. It was suggested by Francis *et al*. that FC treatments offer better IOP control compared to concomitant treatments in real-life settings^20^. Several FCs of commonly employed drugs that lower IOP have been developed in the 2000 s for the purpose of maximising medication compliance in patients^21^. These include combinations of PGA with beta-adrenergic antagonist and beta-adrenergic antagonist with carbonic anhydrase inhibitor or alpha-2-adrenergic agonist. Among the FCs that are recently developed, the combinations of PGA with beta-adrenergic antagonist (PGA/beta-FC) have attracted considerable attention as they combine two separate mechanisms of action. Moreover, both PGA and beta-adrenergic antagonist are frequently employed for glaucoma treatment; therefore, a significant reduction in IOP is expected^22^.

A commonly observed complication with the use of topical drugs to lower IOP in patients with ocular hypertension or glaucoma is the ocular surface disease (OSD). Among glaucoma patients, the OSD prevalence is in the range of 45% to 60%^23–25^. Besides, FCs lower everyday exposure to benzalkonium chloride (BAK) or other preservatives, which are possibly toxic to the ocular surface thereby increasing the treatment side effects^26^. Thus, besides enhancing treatment compliance, FCs are predicted to lower the risks of OSD development.

In Japan, 3 PGA/beta-FCs, tafluprost/timolol FC (TAF/TIM), travoprost/timolol FC and latanoprost/timolol FC (LAT/TIM), have been approved, and latanoprost/carteolol FC (LAT/CAR), which contains carteolol as a beta-adrenergic antagonist other than timolol, is newly approved. We previously reported a greater IOP-lowering effect and lower cytotoxicity with TAF/TIM compared with LAT/TIM^27^. In the current work, we made a comparative assessment of the IOP-lowering effect and toxicity on ocular surface of TAF/TIM versus LAT/CAR, to clarify their efficacy and safety.

## Methods

The IOP-lowering effects were evaluated using cynomolgus monkeys, and ocular surface toxicity was examined in human corneal epithelial cells, which were transformed with SV40. IOP measurements and *in vitro* cytotoxicity study were performed according to the procedures previously described^27^.

### Drugs

TAPCOM combination ophthalmic solution (TAF/TIM; 0.0015% tafluprost, 0.5% timolol) and Tapros ophthalmic solution 0.0015% (TAF; 0.0015% tafluprost) were obtained from Santen Pharmaceutical Co., Ltd. (Osaka, Japan). Mikeluna combination ophthalmic solution (LAT/CAR; 0.005% latanoprost, 2% carteolol hydrochloride) was purchased from Otsuka Pharmaceutical Co., Ltd. (Tokyo, Japan), and Xalatan eye drops 0.005% (0.005% latanoprost) were purchased from Pfizer Inc. (New York, NY, USA).

Carteolol in the estimated formulation of LAT/CAR used in the comparison study of beta-adrenergic antagonist was formulated by Santen Nara Research and Development Center. Estimation of the LAT/CAR formulation was performed using product analysis, confirming that the physicochemical properties, such as viscosity, pH and osmolality, etc., and the ocular penetration of carteolol between LAT/CAR and the estimated formulation were almost the same level.

### Animals

In the present study, we used male adult cynomolgus monkeys, with a body weight of 4.3–10.8 kg (Keari Co., Ltd., Osaka, Japan and Shin Nippon Biomedical Laboratories, Ltd, Tokyo, Japan). The monkeys were housed individually, with the maintenance of a 12-h light–dark cycle. Monkey Bit (Nippon Nosan, Kanagawa, Japan) (100 g per day) and water ad libitum were given to each of the monkeys, along with provisions to enrich the environment such as toys/mirrors, etc. The monkeys were examined once a day by the breeding staff associated with the Animal Care Team at Santen Pharmaceutical Co., Ltd. Temperature and humidity were adjusted to 23 °C (optimal range: 18–28 °C) and 50% (recommended range: 30–70%), respectively, in the breeding environment. Monkeys were acclimatised prior to IOP measurements, to alleviate stress, as follows: acclimation for 1-week in the sitting monkey chair and 3–5 months acclimation for tonometry. Monkeys were administered with local anaesthesia before IOP measurements. Considering that none of the animals suffered from illness, exhibited abnormal health behaviour or died before the completion of the experiments, no medical treatment or humane euthanasia were given to the animals. Following the termination of the study, all the monkeys were taken for use in other ongoing experiments.

All the experimental procedures involving monkeys and their care were performed in compliance with the ARVO Statement for the Use of Animals in Ophthalmic and Vision Research, with the necessary approval and monitoring by the Animal Care and Use Committee at Santen Pharmaceutical Co., Ltd.

### Measurement of IOP and drug administration

In all the animal studies, male, ocular normotensive, cynomolgus monkeys were used. Prior to the study, all the monkeys have undergone training to be restrained in a monkey chair (CL-4535; Primate Products, Miami, FL, USA) and also to go through IOP measurements without the use of sedation or general anaesthesia. In order to measure IOP, each monkey was restrained in the monkey chair, in sitting position, and the topical application of the local corneal anaesthesia was made with 0.4% oxybuprocaine solution (Benoxil ophthalmic solution 0.4%; Santen Pharmaceutical Co., Ltd., Osaka, Japan) to the monkey’s eyes. Then, IOP was measured using a pneumatonometer (Model 30 Classic; Reichert Technologies, Depew, NY, USA). In all the studies, the right eye of each monkey was administered either saline or test drugs between 9:30 AM and 11:00 AM, with the contralateral eye kept untreated.

### IOP-lowering effects of TAF/TIM and LAT/CAR

To evaluate the IOP-lowering effects of TAF/TIM and LAT/CAR, we performed two independent studies. IOP measurements were conducted immediately prior to, and after 2, 4, 6 and 8 h following drug administration in the early term study, whereas in the late term study, these measurements were made just before and after 24, 26, 28 and 30 h following drug administration. Nine monkeys were used in a crossover study design in both studies, such that each monkey participated in all the groups. The average differences in IOP were shown as mean ± standard error of the mean (SEM).

### IOP-lowering effects of TIM and CAR

Measurements of IOP in the monkeys were performed just before and after 2, 4, 6 and 8 h following drug administration. Nine monkeys were used in a crossover study design, such that each animal contributed to all the groups. The average differences in IOP were expressed as mean ± SEM.

### IOP-lowering effects of TAF and LAT

Measurements of IOP were conducted just before and after 24, 26, 28 and 30 h following drug administration. First, nine monkeys were distributed into three groups, with three monkeys assigned to each group based on baseline IOP values. A week later, nine monkeys were divided into three groups, with three monkeys assigned to each group based on baseline IOP values with the animals placed in different groups than the previous week. The average changes in IOP were shown as mean ± SEM.

### Studies on *in vitro* cytotoxicity

#### Testing solutions

Commercially available, undiluted TAF/TIM (TAPCOM) and LAT/CAR (Mikeluna) ophthalmic solutions were directly tested.

#### HCE-T cell culture

Human corneal epithelial cells, immortalised with SV40 (HCE-T; RIKEN BRC, Tsukuba, Japan) were grown in low-glucose Dulbecco’s Modified Eagle Medium (DMEM) plus Ham’s Nutrient Mixture F-12 (DMEM/F12; Life Technologies, Grand Island, NY, USA) containing 15% fetal bovine serum (FBS; Biowest, Nuaillé, France), 5 μg/mL insulin (Akron Biotechnology, LLC, Boca Raton, FL, USA), 40 μg/mL gentamicin (Invitrogen, Carlsbad, CA, USA) and 10 ng/mL epidermal growth factor (EGF; BD Biosciences, San Jose, CA, USA) in a 5% CO_2_ incubator at 37 °C.

#### MTS assay

Cell viability assays were performed employing MTS (3-(4,5-dimethylthiazol-2-yl)-5-(3-carboxymethoxyphenyl)-2-(4-sulfophenyl)-2H-tetrazolium) according to the manufacturer’s instructions (CellTiter 96 AQ_ueous_ One Solution Cell Proliferation Assay, Promega Inc., Madison, WI, USA). This procedure involved culturing HCE-T cells in 96-well plates (seeded at a density of 1 × 10^4^ cells/well) in DMEM/F12 medium with the supplementation of 10% FBS for 48 h. Then, the culture medium was aspirated. Next, DMEM/F12 was used to wash the HCE-T cells once, prior to the addition of test solution (100 μL). Cell viability was assessed at 1, 3 and 5 min of incubation, after removing the test solution, and adding 100 μL of DMEM/F12 and 20 μL of MTS reagent. Absorbance at 490 nm was recorded using a plate reader, to measure viability of the HCE-T cells after incubation for 2 h at 37 °C. Cell viability assays were done in eight replicates in each group, and the results were expressed as percentages of control (DMEM/F12 only) mean values, for each measurement point.

### Statistical analysis

We used EXSUS statistical analysis software, version 8.0.0 (CAC EXICARE Corporation, Tokyo, Japan) to perform all the statistical analyses. All the results are expressed as mean ± SEM. Comparisons among all the groups in both the IOP and *in vitro* cytotoxicity studies were performed using Tukey multiple comparison test.

## Results

### IOP-lowering effect of TAF/TIM compared to LAT/CAR

**Figure 1:**
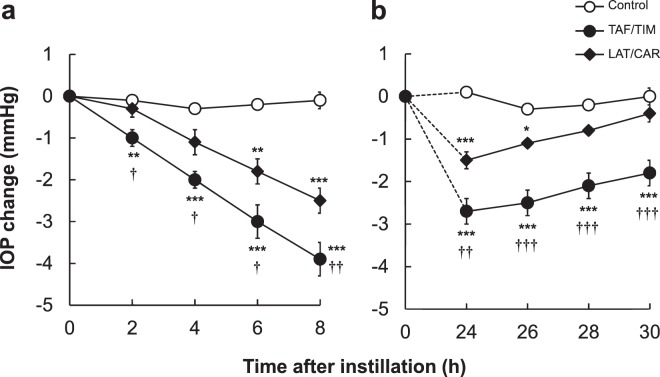
Effects of TAF/TIM and LAT/CAR topical instillation on intraocular pressure (IOP) in ocular normotensive monkeys. Twenty microliters each of either saline (control), TAF/TIM or LAT/CAR were topically administered to the right eyes, keeping the left eyes untreated. Saline was used as a control. Measurements of IOP were performed immediately before and 2, 4, 6 and 8 h following drug instillation (**a**) and immediately before and 24, 26, 28 and 30 h following drug instillation (**b**). IOP changes were determined as the difference from pre-instillation IOP values (i.e., immediately before drug instillation). Data represent the mean ± SEM of nine eyes. ^*^P < 0.05, ^**^P < 0.01, ^***^P < 0.001 vs control, ^†^P < 0.05, ^††^P < 0.01, ^†††^P < 0.001 vs LAT/CAR according to Tukey multiple comparison test.

We performed two independent studies to compare the early (2–8 h) and late (24–30 h) IOP-lowering effects of TAF/TIM and LAT/CAR (Fig. 1). The average initial IOP values in all the groups ranged from 18.8 mm Hg to 19.3 mm Hg in the early term study (Fig. 1a, n = 9) and from 18.7 mm Hg to 19.0 mm Hg in the late term study (Fig. 1b, n = 9). There were no noticeable abnormalities either in general health or ocular condition during the studies.

The IOP-lowering effect of TAF/TIM was seen until 30 h following instillation, and there were significant differences at all the examined time points. LAT/CAR displayed an IOP-lowering effect until 26 h after instillation, with significant changes at 6, 8, 24 and 26 h following instillation.

Comparison of TAF/TIM and LAT/CAR groups revealed a significantly greater IOP-lowering effect by TAF/TIM at all the evaluated time points during 2–8 h. IOP changes in the TAF/TIM and LAT/CAR groups after instillation were as follows: at 2 h, −1.0 ± 0.2 mm Hg and −0.3 ± 0.2 mm Hg; at 4 h, −2.0 ± 0.2 mm Hg and −1.1 ± 0.3 mm Hg; at 6 h, −3.0 ± 0.4 mm Hg and −1.8 ± 0.3 mm Hg; and at 8 h, −3.9 ± 0.4 mm Hg and −2.5 ± 0.3 mm Hg (Fig. 1a). While the effect of IOP-lowering by LAT/CAR was initiated at approximately 4−6 h following instillation, TAF/TIM instillation exerted significant IOP-lowering effect after 2 h and continued thereafter.

Comparison of the IOP-lowering effect duration between the groups revealed TAF/TIM to be superior to LAT/CAR at ≥24 h post-instillation. Thus, the IOP changes in the TAF/TIM and LAT/CAR groups at 24 h were found to be −2.7 ± 0.3 mm Hg and −1.5 ± 0.2 mm Hg; at 26 h, they were −2.5 ± 0.3 mm Hg and −1.1 ± 0.1 mm Hg; at 28 h, they were −2.1 ± 0.3 mm Hg and −0.8 ± 0.1 mm Hg; and at 30 h, they were −1.8 ± 0.3 mm Hg and −0.4 ± 0.2 mm Hg (Fig. 1b). The LAT/CAR mediated IOP-lowering effect was not noticeable at 28 h following instillation, whereas, these effects by TAF/TIM instillation remained even at 30 h.

These results revealed a greater IOP-lowering effect, faster onset and longer duration with TAF/TIM than with LAT/CAR.

### IOP-lowering effect of TIM versus CAR

To clarify the reason for the differences in IOP-lowering effect of TAF/TIM versus LAT/CAR, 0.5% timolol in the TAF/TIM formulation (TIM) and 2% carteolol hydrochloride in the LAT/CAR estimated formulation (CAR) were prepared, and their IOP-lowering effects during 2–8 h were assessed; this period was chosen because the IOP-lowering effect in beta-adrenergic antagonists is thought to present primarily in the early term (2–8 h). PGAs demonstrated a long-lasting IOP-lowering effect in the late term (24–30 h).

**Figure 2:**
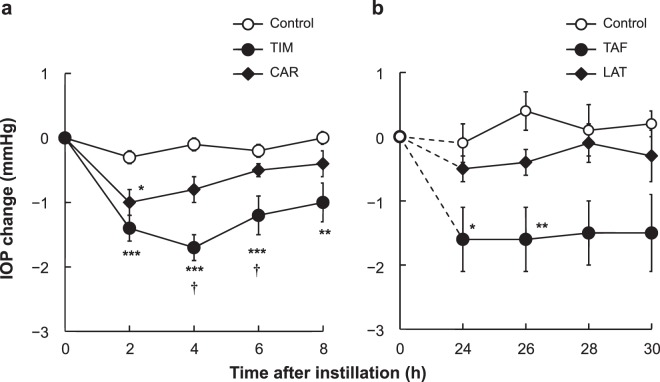
Effects of topical instillation of each composition of TAF/TIM and LAT/CAR on intraocular pressure (IOP) in ocular normotensive monkeys. Twenty microliters each of either saline, TIM, CAR, TAF or LAT were instilled topically in the right eyes, keeping the left eyes untreated. Saline was used as a control. Measurements of IOP were performed immediately before and 2, 4, 6 and 8 h following drug instillation, in comparison with TIM and CAR (**a**), and immediately before and 24, 26, 28 and 30 h following drug instillation in comparison with TAF and LAT (**b**). IOP changes were determined as the difference from pre-instillation IOP measurements. Results represent mean IOP change ± SEM of nine eyes (**a**) and six eyes (**b**), respectively. ^*^P < 0.05, ^**^P < 0.01, ^***^P < 0.001 vs control, ^†^P < 0.05 vs CAR according to Tukey multiple comparison test.

Figure 2a shows changes in IOP. The mean values of initial IOP were 17.2 mm Hg to 17.3 mm Hg (n = 9). There were no noticeable abnormalities either in general health or ocular condition during the present study.

The IOP-lowering effect of TIM was significant at 2 h after instillation, and it continued thereafter; the IOP-lowering effect of CAR was seen until 4 h following instillation, with major differences noticeable at 2 h post-instillation. There was no demonstrable IOP-lowering effect by CAR at 6 h but such effect remained for TIM at 8 h after instillation.

When comparing the TIM and CAR groups, TIM displayed a greater IOP-lowering effect compared to CAR, with significant differences noticed at 4 h and 6 h following instillation. The IOP changes in the TIM and CAR groups were −1.4 ± 0.2 mm Hg and −1.0 ± 0.2 mm Hg at 2 h; −1.7 ± 0.2 mm Hg and −0.8 ± 0.2 mm Hg at 4 h; −1.2 ± 0.3 mm Hg and −0.5 ± 0.1 mm Hg at 6 h; and −1.0 ± 0.3 mm Hg and −0.4 ± 0.2 mm Hg at 8 h after instillation.

These results demonstrated that TIM had a greater IOP-lowering effect and faster onset over CAR.

### IOP-lowering effect of TAF compared to LAT

To clarify the reason for the differences in the IOP-lowering effect of TAF/TIM and LAT/CAR, particularly their long-lasting effects in the late term (24–30 h), we evaluated the effect of 0.0015% tafluprost (TAF; 0.0015% Tapros ophthalmic solution) and 0.005% latanoprost (LAT; 0.005% Xalatan eye drops) during 24–30 h.

Figure 2b shows changes in IOP. The mean initial IOP values were 18.4 mm Hg to 18.7 mm Hg (n = 6). There were no significant abnormalities either in ocular condition or in general health during the study.

The IOP-lowering effect of TAF lasted until 30 h following instillation and at 24 h and 26 h, there were significant differences. However, IOP-lowering effect was not seen with LAT at any of the evaluated time points.

TAF revealed a greater IOP-lowering effect compared to LAT at all the evaluated time points. IOP changes in the TAF and LAT groups were −1.6 ± 0.5 mm Hg and −0.5 ± 0.2 mm Hg at 24 h, −1.6 ± 0.5 mm Hg and −0.4 ± 0.2 mm Hg at 26 h, −1.5 ± 0.5 mm Hg and −0.1 ± 0.3 mm Hg at 28 h and −1.5 ± 0.6 mm Hg and −0.3 ± 0.4 mm Hg at 30 h following instillation. LAT-mediated IOP-lowering effect was almost unnoticeable at 24 h post-instillation but was still observed in TAF group at 30 h following instillation.

These findings demonstrated a greater IOP-lowering effect and longer duration with TAF compared to LAT.

### Comparison of cytotoxicity on HCE-T cells by TAF/TIM and LAT/CAR

**Figure 3:**
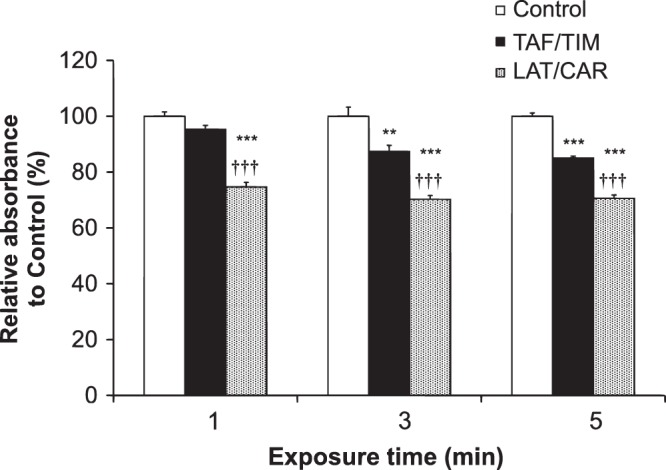
Cytotoxicity of TAF/TIM and LAT/CAR on HCE-T cells. MTS cell viability assays were conducted at 1, 3 and 5 min of incubation. Controls were setup with only medium (DMEM/F12). Control was considered as 100% and the treated cell viability was shown as a percent of control. Results are mean ± SEM of eight replicates. ^**^P < 0.01, ^***^P < 0.001 vs control, ^†††^P < 0.001 vs TAF/TIM according to Tukey multiple comparison test.

Following exposure of HCE-T cells for 1, 3 and, 5 min to drugs, cell viability was significantly decreased by LAT/CAR at all the time points in comparison to the control cells: 74.7% ± 1.6% at 1 min; 70.2% ± 1.4% at 3 min and 70.6% ± 1.2% at 5 min. Conversely, no significant decrease was seen in TAF/TIM at 1 min exposure (95.3% ± 1.4%), but significant decreases were found at 3 and 5 min exposure (87.4% ± 2.2% and 85.1% ± 0.7%, respectively), though these decreases were much less compared to those for LAT/CAR (Fig. 3).

TAF/TIM had significantly lower cytotoxicity compared to LAT/CAR at all the evaluated time points.

## Discussion

TAF/TIM is approved in many countries as a PGA/beta-FC. LAT/CAR was recently approved in Japan, and in 2000, LAT/TIM was the first approved PGA/beta-FC in Sweden. We previously reported that TAF/TIM had a greater IOP-lowering effect and lower cytotoxicity compared to LAT/TIM^27^. The difference between LAT/TIM and LAT/CAR is due to the difference between the beta-adrenergic antagonists timolol and carteolol. In a previous report, it was revealed that timolol had a greater effect of IOP-lowering compared to carteolol^28^, suggesting that LAT/TIM exhibits a greater IOP-lowering effect compared to LAT/CAR. Therefore, TAF/TIM was considered to be superior to LAT/CAR because it exhibited a better IOP-lowering effect than that exhibited by LAT/TIM.

In the present study, we evaluated the potency and duration of the IOP-lowering effects of TAF/TIM and LAT/CAR (Fig. 1). The study was performed following a single instillation. The intervals between instillations were one week apart so that there is a wash-out period to avoid carryover of the drug among each term of the crossover studies. After it was confirmed that IOP returned to the baseline value after one week (Figs 1 and 2), the next term of the study was subsequently performed. In our previous report^27^, it was performed with the same schedule as that of the studies evaluating the IOP-lowering effects of TAF/TIM and latanoprost/timolol FC in a crossover fashion. Accordingly, it was expected that the experiment was appropriately performed without drug carryover. LAT/CAR is a newly approved FC in Japan; therefore, there are no reports comparing LAT/CAR and existing PGA/beta-FCs. In the present study, we demonstrated a greater IOP-lowering effect as well as faster onset and longer duration in TAF/TIM compared with LAT/CAR, indicating the superior efficacy of TAF/TIM. The differences in the IOP-lowering effect were estimated to be a result of the differences of each monopreparation, timolol and carteolol, and tafluprost and latanoprost in the present study. The early and late term differences in efficacy were attributable to the differences between timolol and carteolol and between tafluprost and latanoprost, respectively.

We evaluated the strength of the IOP-lowering effects of TIM and CAR in the present study (Fig. 2a) and displayed a stronger IOP-lowering effect and faster onset in TIM compared to CAR in the early term (2–8 h), indicating superior efficacy of TIM compared to CAR. Consequently, we also examined the strength and duration of the IOP-lowering effects mediated by TAF and LAT (Fig. 2b). In this experiment, we showed that TAF had a greater IOP-lowering effect and longer duration compared to LAT, indicating superior efficacy of TAF in comparison to LAT. These results revealed that the differences in the IOP-lowering effect were due to the differences between timolol and carteolol as a beta-adrenergic antagonist and considered to be mainly attributable to efficacy in the early term and onset, and the differences between tafluprost and latanoprost as a PGA and considered to be mainly attributable to efficacy in the late term and duration.

In the present study, we used ocular normotensive monkeys for evaluating the IOP-lowering effects of PGAs (tafluprost and latanoprost), beta-adrenergic antagonists (timolol and carteolol), and their FCs. When considering extrapolating the results in monkeys to humans, we should consider the difference between monkeys and humans in the aqueous humor dynamics, which is thought to regulate IOP. Aqueous humor drains approximately 80%–90% from the trabecular outflow pathway in aged humans^29^; however, it is about 50% in young humans^29^ and monkeys^30–32^. According to the similarity in the aqueous humor dynamics between young humans and monkeys, the results from young monkey may directly predict those in young humans. Conversely, it is difficult to predict the IOP-lowering effects in patients with glaucoma, which are almost ageing human, because the IOP-lowering effects in ageing monkey have not been evaluated in the present study. When considering drug responses, it has been confirmed that tafluprost, latanoprost, timolol and carteolol show IOP-lowering effects in humans^28,33–35^. Similar to that in humans, the IOP-lowering effects of these drugs, except for carteolol, have been confirmed in monkeys^9,36^, and the present study showed the IOP-lowering effect of carteolol in monkeys (Fig. 2), suggesting that tafluprost, latanoprost, timolol and carteolol have good correlations in humans and monkeys. Therefore, it may be expected that TAF/TIM is better than LAT/CAR with regard to the IOP-lowering effect in humans, as observed in monkeys in the present study.

A single instillation of TAF/TIM lowered the IOP for >30 h. Reduction in IOP is less influenced by neglecting the administration of drugs to lower IOP, showing better efficacy with duration. The long-lasting action implies that missing a dose of the drug occasionally may not lead to substantial fluctuations in IOP. Reportedly, such aberrations in IOP are a risk factor for the progression of glaucoma^37^. Thus, the long-lasting effect of IOP reduction by TAF/TIM may help in stably controlling IOP and in slowing the progression of glaucoma. The results of the present study suggest that TAF/TIM is superior to LAT/CAR with respect to its IOP-lowering effect.

In the present study, the effect of the 2 FCs, TAF/TIM and LAT/CAR, was also compared on the ocular surface health, by assessing *in vitro* cytotoxicity. Chronic application of topical anti-glaucoma medications causes side effects viz., allergy, ocular inflammation, failed filtration surgery and dry eye syndrome^38–40^, probably due to the components and additives of the medications. Therefore, there exists an immediate need clinically for IOP-lowering drugs that lack corneal toxicity or with significantly reduced side effects. In the current study, TAF/TIM exhibited much lower cytotoxicity compared to LAT/CAR (Fig. 3). We considered that the toxicity of additives such as surfactants, chelating agents and preservatives, such as BAK and borate, may affect cytotoxicity. TAF/TIM includes a low concentration of BAK (0.001%), which reportedly does not affect cell viability^41^, and LAT/CAR includes borate as a preservative. The lower cytotoxicity of TAF/TIM is probably due to the differences in the preservatives, as compared to LAT/CAR. These results suggest that TAF/TIM may lead to lower ocular surface damage unlike the LAT/CAR in clinical use. Also, the superiority of TAF/TIM is probably related to the low concentration of BAK in the formulation. It is important to keep in mind that this result and suggestion is based on *in vitro* assays. Further studies evaluating the effects of these drugs in animals are needed to ascertain their relevance in clinical use.

In conclusion, results from the *in vivo* and *in vitro* studies in the present work show that TAF/TIM is superior to LAT/CAR with regard to the IOP-lowering effect and ocular surface toxicity.

## Data Availability

All the datasets from the present study may be obtained from the corresponding author upon request.
